# An HIV Epidemic Model Based on Viral Load Dynamics: Value in Assessing Empirical Trends in HIV Virulence and Community Viral Load

**DOI:** 10.1371/journal.pcbi.1003673

**Published:** 2014-06-19

**Authors:** Joshua T. Herbeck, John E. Mittler, Geoffrey S. Gottlieb, James I. Mullins

**Affiliations:** 1 Department of Microbiology, University of Washington, Seattle, Washington, United States of America; 2 Department of Medicine, University of Washington, Seattle, Washington, United States of America; Imperial College London, United Kingdom

## Abstract

Trends in HIV virulence have been monitored since the start of the AIDS pandemic, as studying HIV virulence informs our understanding of HIV epidemiology and pathogenesis. Here, we model changes in HIV virulence as a strictly evolutionary process, using *set point viral load* (SPVL) as a proxy, to make inferences about empirical SPVL trends from longitudinal HIV cohorts. We develop an agent-based epidemic model based on HIV viral load dynamics. The model contains functions for viral load and transmission, SPVL and disease progression, viral load trajectories in multiple stages of infection, and the heritability of SPVL across transmissions. We find that HIV virulence evolves to an intermediate level that balances infectiousness with longer infected lifespans, resulting in an optimal SPVL∼4.75 log_10_ viral RNA copies/mL. Adaptive viral evolution may explain observed HIV virulence trends: our model produces SPVL trends with magnitudes that are broadly similar to empirical trends. With regard to variation among studies in empirical SPVL trends, results from our model suggest that variation may be explained by the specific epidemic context, *e.g.* the mean SPVL of the founding lineage or the age of the epidemic; or improvements in HIV screening and diagnosis that results in sampling biases. We also use our model to examine trends in community viral load, a population-level measure of HIV viral load that is thought to reflect a population's overall transmission potential. We find that community viral load evolves in association with SPVL, in the absence of prevention programs such as antiretroviral therapy, and that the mean community viral load is not necessarily a strong predictor of HIV incidence.

## Author Summary

Virulence can be defined as disease severity; virulence of a pathogen may evolve as the rates of host mortality and transmission are balanced. HIV virulence trends are estimated using set point viral load, a proxy for the rate of HIV disease progression. To assess the capacity for HIV virulence to evolve and to place published virulence trends in an evolutionary context, we developed an evolutionary model based on HIV viral load dynamics. Our model reveals that HIV virulence evolves to an optimal set point (∼4.75 log10 copies/mL) similar to levels seen in natural populations. In comparing published trends to model-based trends, we infer: a) published trends are consistent with HIV adaptation to the human population; and b) variation among published trends may be explained by epidemic context or by sampling biases resulting from improvements in HIV screening and diagnosis. We also assess trends in HIV community viral load, defined as the mean viral load of all reported viral loads in a community, and thought to reflect a population's transmission potential. We find that community viral load evolves in association with set point, in the absence of prevention programs like antiretroviral therapy, and does not necessarily predict HIV incidence.

## Introduction

Virulence can be defined as the severity of disease caused by a pathogen; the virulence of a pathogen may evolve within a host population as the rates of transmission and host mortality are balanced by natural selection. For HIV, virulence can be defined as the rate of disease progression in the absence of antiretroviral treatment. Understanding if HIV virulence has evolved will inform our understanding of HIV epidemiology and pathogenesis, as increases in HIV virulence would result in more rapid disease progression [1]–[4], the earlier initiation of antiretroviral therapy [5], and an increased per-contact transmission risk [6]–[10].

The estimation of epidemic trends in HIV virulence includes the measurement and analysis of proxy markers for HIV disease progression, with set point viral load (SPVL) the most prognostic single marker of the time to AIDS after HIV infection [1]–[4]. While exact definitions of SPVL vary, it is generally the HIV plasma RNA viral load after the resolution of acute infection, but within 6 months to two years after seroconversion (and prior to initiation of antiretroviral therapy). We previously completed a meta-analysis of 8 published studies of population-level trends in SPVL; our meta-analysis found a positive summary trend (0.013 log_10_ copies/mL/year, *P* = 0.07) [11], consistent with increased virulence of HIV. However, the studies also showed large variation in SPVL trends (range: −0.013 to 0.035 log_10_ copies/mL/year) [11].

The causes of this variability remain unexplained, but may include estimation methods (clinical or statistical) or differences among the HIV cohorts (and associated local epidemics) in which trends were studied. These cohorts contain primarily men of European descent with HIV subtype B infections. Our previous analysis of study parameters in these 8 cohorts (including transmission risk group frequency, sample size, length, calendar year time period, seroconversion lag, and sampling lag) showed that only seroconversion lag was associated with SPVL trend; *i.e.*, shorter periods between the last negative and first positive HIV-1 antibody tests were correlated with increased SPVL trends. It followed that for the 6 of 8 studies in the meta-analysis that were prospective seroconverter cohorts, the summary SPVL trend was 0.018 log_10_ copies/mL/year, 38% greater than the summary trend of 0.013 copies/mL/year for all eight cohorts (including both seroconverter and seroprevalent cohorts). However, this explanation for variation in SPVL trends is insufficient, as trends from seroconverter cohorts are still highly variable (range: −0.002 to 0.035 log_10_ copies/mL/year) [11].

Positive trends in mean population SPVL (HIV virulence) may reflect the process of viral adaptive evolution in the human population; recent modeling studies [12], [13] have proposed the existence of an evolutionarily optimal SPVL of 4.52 log_10_ copies/mL, defined as the viral load that balances transmission probability with infected lifespan. For example, low SPVL will result in lower infectivity but more total transmissions (due to greater life expectancy), whereas high SPVL will result in high infectivity but fewer total transmissions (due to decreased life expectancy). The proposed optimal SPVL of 4.52 log_10_ was qualitatively consistent with mean SPVL levels found in the Amsterdam Cohort Study (4.36 log_10_ copies/mL) [12] and the Zambian Transmission Study (4.74 log_10_ copies/mL) [9]. Mean SPVLs in the studies included in our meta-analysis were also qualitatively similar to 4.52, albeit with a wider range (from 4.25 to 5.2 log_10_ copies/mL). An even greater range in population viral loads was seen in a review of 57 studies, where medians ranged from 3.7 and 5.6 log_10_ copies/mL and the overall median was 4.45 log_10_ copies/mL [14]. These 57 studies were not studies of SPVL distributions, specifically; however, viral loads in the asymptomatic period are generally stable and likely to be close to SPVL [15], [16].

We have developed a stochastic, agent-based HIV evolutionary and epidemic model based on the dynamics of HIV viral load. This model is unique for HIV epidemic models in that it allows for the viral virulence phenotype (set point viral load) to change over the course of an epidemic. The model contains known functions related to viral load trajectories in acute, chronic and disease stages, viral load and transmission probability, SPVL and disease progression rate, and the heritability of SPVL across transmission pairs. These components provide an evolutionary framework in which a balance is achieved between efficient transmission and slow disease progression. This type of evolutionary structure for HIV transmission potential was proposed by Fraser [12].

Our primary aims are to understand both the underlying causes of empirical SPVL trends and also the variation among populations/cohorts in the estimation of these trends (rather than variation in SPVL among individuals *e.g.*, [15], [17], or the relative contributions of viral genetic and environmental factors to SPVL variation [13]). To do this, we estimate the temporal changes in SPVL that can result from adaptive viral evolution in human populations, and we assess deviations from these trends related to virologic parameters such as initial mean SPVL, maximum per-contact transmission rate, or epidemic stage. We also examine the effects of potential sampling biases due to improvements in the rates of HIV diagnosis, changes in cohort recruitment procedures, or earlier initiation of ART after diagnosis—biases that were not explicitly considered by the studies of empirical SPVL trends described in the meta-analysis.

A secondary aim is to understand trends in HIV community viral load (CVL). CVL is typically defined as the arithmetic or geometric mean (or median) of all reported individual viral loads (individuals diagnosed and sampled, with detectable viral load) in a specific community. CVL is considered to be a heuristic proxy for overall transmission potential (and level of HIV-associated health care) in a given community [18]–[21]. Thus, the ability to accurately assess trends in CVL is critical for prevention programs that promote reduced population viral load and transmission potential as a measure of effectiveness: CVL needs to be a robust and informative metric for comparisons through time, meaningfully related to HIV epidemiological parameters that are important to the public health community. However, recent work has described potential pitfalls in the relationship between CVL and transmission potential (*e.g.*, issues of sampling bias, population context, and ecological fallacy) [22]. In our analysis, we focused on the potential evolutionary context of trends in CVL: there are similarities between the estimation of SPVL and CVL trends, and we hypothesize that understanding biases related to SPVL trends will inform our understanding of CVL trends. Overall, we expect our model will improve our understanding of HIV epidemics and the virologic metrics that are used to study them.

## Materials and Methods

We have constructed a stochastic agent-based HIV epidemic model that simulates viral load dynamics within and between individuals. The model has four main components: 1) the distribution of set point viral load (SPVL) in a population of HIV-infected individuals linked via a sexual contact network (the population component) [12], [14], [23]; 2) the predictive relationship between HIV viral load and the per-contact viral transmission rate [6]–[10]; 3) the predictive relationship between SPVL and the rate of disease progression [1]–[4], [16]; and 4) the partial heritability of SPVL across transmission pairs (the viral genotype plays a role in determining SPVL) [24]–[27]. These components were embedded within a simple model for a sexual contact network. The model includes fixed parameters (with estimates following from previous studies, when possible) and variable parameters (with unknown or uncertain estimates that we varied to evaluate their effects on epidemic and evolutionary output) (Table 1). The underlying model was written in C with a front-end written in R.

**Table 1:** Parameters of the model and standard initial values.

Parameter	Value
Initial overall population size	75000
Initial number of infected	500
Average number of partners per person	0.9
Minimum duration of any relationship, days	1.0
Maximum duration of any relationship, days	1.0
Probability that a couple will have sex, per day	1.0
Maximum number of concurrent partners	1.0
Natural death rate, per day	0.0001
Maximum transmission rate, per day, asymptomatic stage	0.0025 [12], [13]
Viral load at half maximum transmission rate, copies/mL	13938 [12], [13]
Hill coefficient, transmission rate	1.02 [12], [13]
Shape parameter, transmission rate	3.46 [12], [13]
Maximum time to AIDS, days	9271 [12], [13]
Time to half maximum time to AIDS period, days	3058 [12], [13]
Hill coefficient, time to AIDS	0.41 [12], [13]
Viral load at time zero, copies/mL	100
Viral load at peak viremia, copies/mL	1.0×10^7^ [6], [60]
Time to peak viremia, days	21 [6], [60]
Total time of acute infection, days	91
Total time of AIDS before death, days	91
Viral load at AIDS, copies/mL	5.0×10^6^
Average SPVL at time zero, log_10_ copies/mL	4.5 [12], [13]
Variance of log_10_ SPVL	0.8 [14]
Mutational variance, annual	0.01
Viral load progression rate, natural log, annual	0.01 [15], [16]
Heritability of SPVL across transmissions	0.5 [25], [26]

### Distribution of set point viral load within a population

This component includes HIV-uninfected people, HIV-infected people, and people who have died of AIDS. Each simulation starts with *N* total HIV-uninfected and infected individuals at time zero, with each infected individual (of *n* total HIV-infected individuals) provided a SPVL value following from a Gaussian distribution with user-defined mean and variance, with the variance parameter following published estimates [12], [14]. Entry of new (uninfected) individuals into the population is at a constant input rate such that the population, in absence of HIV infection, will stay at its initial value. Infected individuals may die of their HIV infections according to formulas given below. Further model details are given in Text S1.

### Epidemic simulations

We ran epidemic simulations for 100 years, in discrete time-steps of one day. For each model run we tracked individual viral load trajectories, infected lifespan, sexual partnerships and transmission histories, and epidemic growth or decline, and we estimated population measures of AIDS mortality, SPVL trends and heritability. The mean, median, and variance of SPVL in the population could be calculated directly at any day in a simulated epidemic. Likewise, the mean, median, and variance of community viral load (CVL) could be calculated at any day using the viral load from each currently alive and infected individual. We conducted sensitivity analyses on each parameter from Table 1, but focused on: the rate of viral load increase in the asymptomatic period (*s* from Equation 1 in Text S1); the rate of disease progression (maximum time to AIDS after infection, *D*_max_ from Equation 2 in Text S1); and the maximum daily rate of transmission in the asymptomatic period (*B*_max_ in Equation 3 in Text S1).

To examine the potential for adaptive evolution of HIV virulence, we examined 10 replicate epidemic simulations for three different values of initial population mean SPVL (3.5, 4.5, and 5.5 log10 copies/mL). Trends in SPVL were calculated by regressing against the times of infection recorded for each individual (each individual had individual-specific SPVL and time of infection).

### Null distributions of 20-year SPVL trends

We sought to place published SPVL trends in the context of results from our epidemic model. To do this, we created hypothetical null distributions of SPVL trends for epidemics with initial mean SPVL values equal to 3.5, 4.5, or 5.5 log_10_ copies/mL. For each of the 10 replicate runs for each initial mean SPVL, we randomly chose 100 separate 20-year time periods with replacement; we chose 20-year periods as most empirical estimates of SPVL trends cover 20-year periods. From each of these 20-year time periods we estimated a univariate linear regression of SPVL by calendar year of infection. This created a distribution of 1×10^3^ possible observed SPVL trends (100 20-year periods from 10 replicate model runs) for any given set of model parameters, where only the starting random number seed and the randomly chosen 20-year period were different between trend estimates.

The HIV epidemics of European and North American countries are younger than 100 years; the first introduction of HIV to the US is estimated to have occurred in 1969 [28] with introductions to Europe following. Empirical SPVL trends have been estimated most often using data from approximately 1985 to 2010 [29]. This may affect our choice of an appropriate null distribution for SPVL trends produced by our model. To accommodate this uncertainty we created separate null distributions, each spanning a different subset of the complete 100-year simulated epidemics: a) all 100 years of the model output; b) years 10–100 of the model output, under the assumption that European and North American subtype B epidemics began ∼1970, which, as studies of empirical SPVL trends began sampling at the earliest in 1984, leaves a ∼10-year window of the HIV epidemic that was not sampled by cohorts; c) years 0–40 of the model output, under the assumption that because empirical studies of SPVL trends include years up to ∼2010, this represents the first 40 years of the subtype B epidemic (∼1970 to 2010); and d) years 10–40, the most restrictive null, meant to reflect the empirical sampling years ∼1980 to 2010.

### Recreation of sampling biases

Two distinct types of sampling biases may potentially result from the fact that HIV viral loads in primary infection are higher in symptomatic individuals [30], [31]: a trend in mean SPVL may result from improvements over time in referral or cohort recruitment practices [32], or improvements over time in diagnostic techniques and identification of new HIV infections [33]. For example, if rapid progressors are more readily identified and diagnosed as the epidemic progresses, and these individuals initiate ART before set point is measured, then fewer individuals with (relatively) high SPVL will be sampled as the epidemic ages. This is a bias caused by earlier diagnosis and ART initiation, and may lead to inferred trends of decreasing virulence. Alternatively, if increased diagnosis and recruitment of newly infected individuals is associated with improvements in referral or cohort recruitment, but does *not* lead to earlier ART initiation, biased sampling in the opposite direction may result. In this case, relatively more symptomatic individuals would be sampled, and trends of increasing virulence may be inferred. (We recognize that increased and earlier diagnosis not leading to earlier ART initiation is not consistent with current clinical practice; however, most published studies of HIV virulence trends included sampling years from the 1980s and 1990s.)

We recreated the above types of biased sampling in simulated epidemics as follows: 1) we divided randomly chosen 20-year epidemic periods (used to estimate SPVL trends) into individuals infected in years 1–10 versus years 11–20; 2) from the latter subpopulation (years 11–20), we created subsamples that included individuals with SPVLs either greater or less than SPVL = 5.0 log_10_ copies/mL (http://aidsinfo.nih.gov/guidelines/archive/adult-and-adolescent-guidelines); 3) depending on the type of sampling bias to be recreated, we randomly selected a portion of individuals to be removed from either the “SPVL>5.0” or “SPVL<5.0” subsamples (Figure S1).

### Community viral load trends

For each day in a simulated epidemic, we calculated community viral load (CVL) as the mean and median viral load of all HIV-infected individuals who were currently in day 45 or greater of their infection (one half of the time to reach set point; with the length of acute infection set to 90 days in our standard parameter settings). This threshold is meant to mimic empirical estimates of CVL, where the majority of individuals in early acute infection are unsampled and do not contribute to CVL estimates.

## Results

We developed a stochastic, agent-based, HIV evolutionary and epidemic model based on viral dynamics. Within this model, variation in SPVL across individuals is due to viral and other (host and environmental) factors. This provides an evolutionary framework based on viral transmission potential [12], [13] that determines the population trends in virulence and results in an evolutionary balance between virulence (disease severity) and transmission, as SPVL governs the rate of disease progression (the length of the asymptomatic period) and the transmission rate (based on individual viral load, which follows from SPVL as infections progress). Our primary goal is to understand the underlying causes of empirical SPVL trends and also the variation among cohorts/populations in the estimates of these SPVL trends.

### Model validation

**Figure 1:**
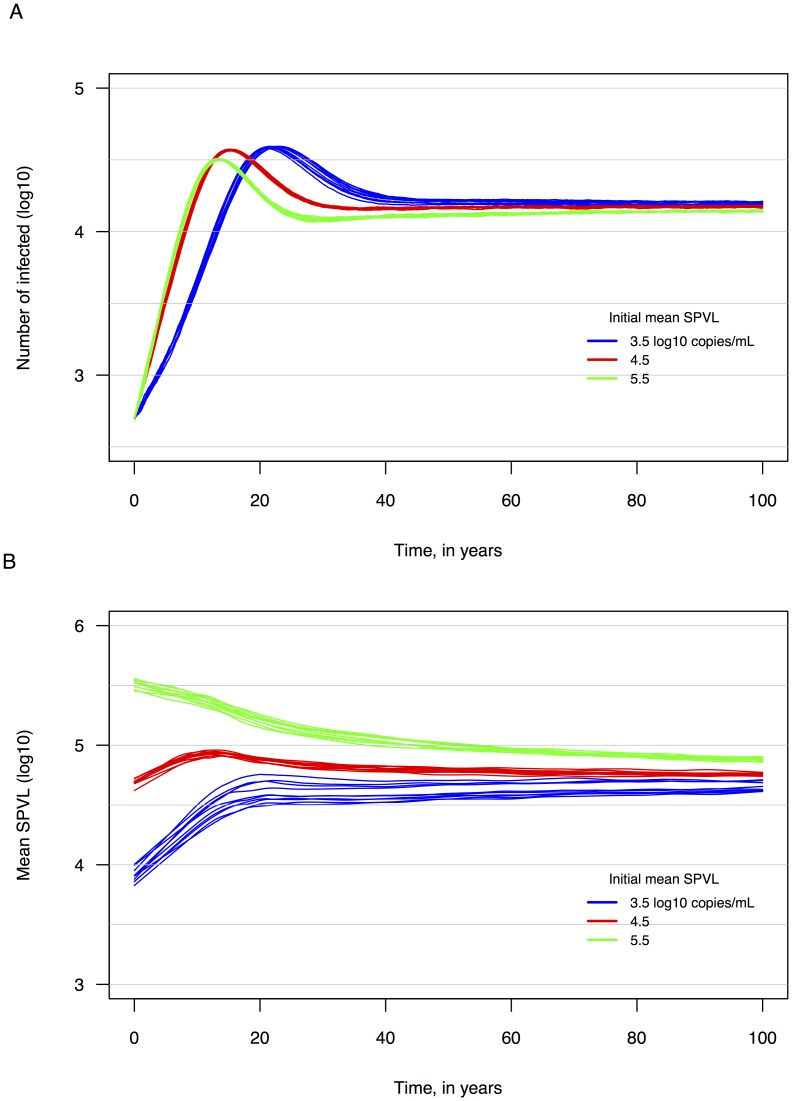
Variation across replicate simulated epidemics. **A.** Epidemic size over time. Epidemic runs with each initial SPVL were repeated 10 times, each run with a different random number seed. **B.** Population mean set point viral load (SPVL; log_10_ HIV RNA copies/mL at the end of acute infection) over time, using a locally weighted polynomial regression curve (Lowess fit = 0.1). Mean SPVL evolves toward 4.75 log_10_ copies/mL.

For model runs of 100 years with standard parameter values (Table 1), we found the following: epidemic growth, starting from 500 HIV-infected individuals in an initial population size of 75,000 (infected and uninfected), varies across initial mean SPVL values. Runs with intermediate (4.5) and high (5.5) initial mean SPVLs resulted in faster rates of epidemic growth; these resulted in >35,000 HIV-infected individuals before year 20 (Figure 1A). Kaplan-Meier survival curves stratified by quartiles of SPVL were consistent with published survival analyses from two separate cohorts [3], [12] (Figure S2). The estimated heritability of SPVL across transmission pairs were consistent with empirical estimates of SPVL heritability [24]–[27], and decreased over the course of simulated epidemics as variance in SPVL decreased (Figure S3). Population variation in SPVL and VL were consistent with empirical estimates: a recent meta-analysis reported interquartile ranges (IQR) for 51 studies of population viral loads [14] with an average IQR of ∼1.0 log_10_ copies/mL; IQRs of SPVL for our model were 0.9, 0.86, and 0.84 log_10_ copies/mL for new infections taking place in years 25, 50 and 75 of simulated epidemics, respectively (Figure S4).

### Frequency of transmissions from early HIV infection

For a standard epidemic run (Table 1), ∼5% of all infections (over 100 year epidemic simulations) took place while the source partner was in “early HIV infection” (defined as 3 months, up to Fiebig stage V [34]), although this frequency was higher (∼10%) in the early stages of simulated epidemics (Figure S5). Changing the definition of “early HIV infection” to include the entire first year of infection increased the frequency of early infection transmissions to ∼20% of all transmissions (and this frequency is higher, ∼25%, if only simulated epidemic years 10 to 40 (∼1980 to 2010) were considered) (Figure S5).

### Set point viral load evolves to an optimal level

We found that SPVL evolves toward a equilibrium value of approximately 4.75 log_10_ copies/mL, regardless of the mean SPVL of the founding population of infected individuals (at time zero) (Figure 1B). Variability across 10 replicate model runs (with only the random number seed changed) in SPVL trends was minimal (Figure 1B). This SPVL is slightly higher than the 4.52 optimal SPVL predicted by Fraser [12] and Shirreff [13], as well as the 4.45 overall median of 57 studies reported by Korenromp [14].

### Empirical trends are similar to model-based trends

**Figure 2:**
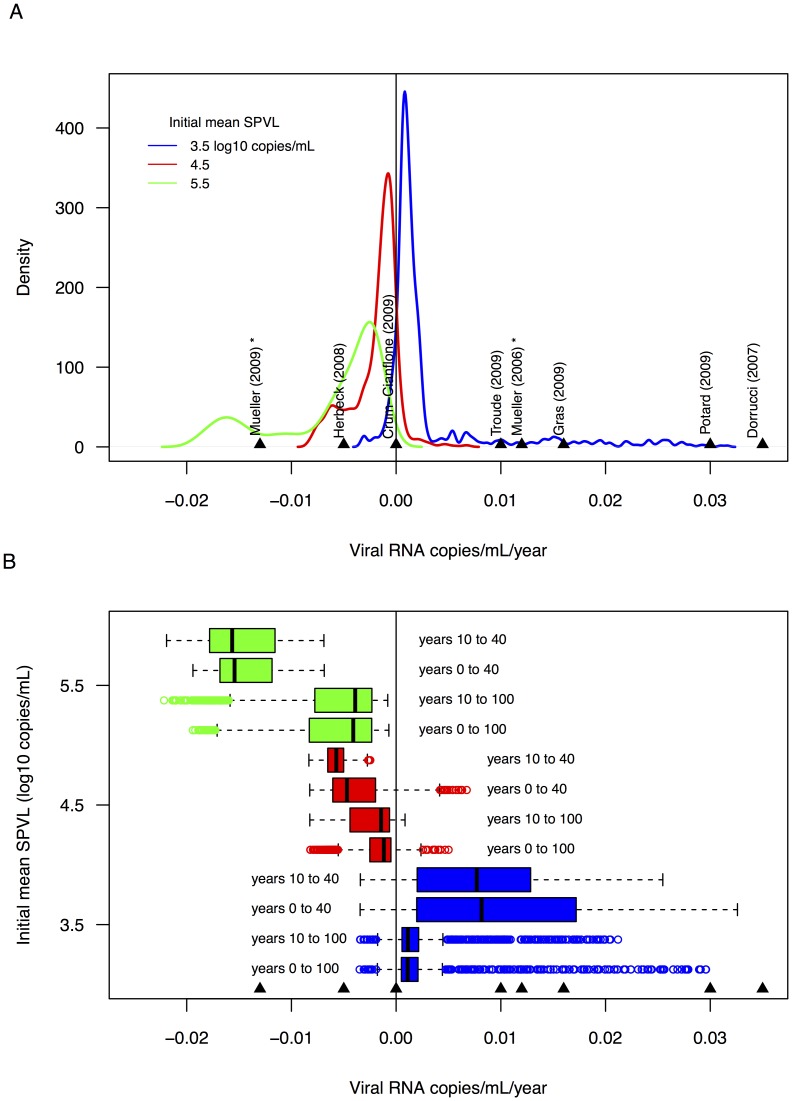
A. Empirical SPVL trends overlaid onto distributions of simulated 20-year trends. Distributions of linear SPVL trends (log_10_ HIV RNA copies/mL/year) were estimated from 100 randomly sampled 20-year time periods for 10 replicate simulations for each initial mean SPVL = 3.5, 4.5 or 5.5 log_10_ HIV RNA copies/mL (creating 1000 total 20-year trends for each initial mean SPVL). Empirical (published) annual linear SPVL trends are overlaid (arrows and references). References with asterisks are seroprevalent cohorts; all others are seroconverter cohorts. **B.** **Selection of an appropriate null changes the distribution of simulated SPVL trends.** Separate null distributions, each spanning a different subset of the complete 100-year simulated epidemics: all 100 years of the model output; years 10–100 of the model output, as European and North American subtype B epidemics began ∼1970, and studies of empirical SPVL trends began sampling at the earliest in 1984, leaving a ∼10-year window of the HIV epidemic not sampled by the cohorts; years 0–40 of the model output, as the empirical studies of SPVL trends include years up to ∼2010, so this represents the first 40 years of the subtype B epidemic (∼1970 to 2010); and years 10–40, reflecting the empirical sampling years ∼1980 to 2010.

We next compared empirical SPVL trends to results from our model by producing null distributions of 20-year linear SPVL trends. Figure 2A shows the different null distributions for model runs with founder mean SPVLs of 3.5, 4.5, and 5.5 log_10_ copies/mL, using all 100 years of simulated epidemics. Broadly, trends estimated from epidemics with initial mean SPVL = 3.5 were positive and trends from epidemics with initial mean SPVL = 4.5 or 5.5 were negative (Figure 2A). Interestingly, the distribution for epidemics with starting mean SPVL = 4.5 included mostly negative trends despite the optimal SPVL equal to ∼4.75 copies/mL, due to transient increases in virulence early in epidemics, before the number of susceptible individuals begins to decrease (Figure 1B) [13], [35]. We compared the empirical SPVL trends to these null distributions, and the magnitudes of the empirical trends were similar to (and therefore consistent with) the model-based trends. The majority of these trends were located to the far right (positive) side of the distributions (SPVL trends>0.01 log10 copies/mL/year) (Figure 2A). (95% confidence intervals for the empirical trends are overlaid on null distributions created from model years 0 to 100 in Figure S6A.)

### Founding HIV lineages may have had low virulence

When we compared empirical SPVL trends to potentially more appropriate null distributions (produced by sampling only years 0 to 40, or years 10 to 40, of simulated epidemics), the empirical trends were even more similar to model-based trends. Specifically, the larger empirical trends (>0.01 log10 copies/mL/year) were consistent with the median trends from null distributions for simulated epidemics with initial mean SPVL = 3.5 (Figure 2B). Thus, the larger empirical trends may be unbiased measures of adaptive viral evolution in populations where the initial founding viral lineages contained low virulence. The median SPVL trend for the null distribution produced from sampling from years 10 to 40 of epidemics with initial mean SPVL = 3.5 was 0.0073 log_10_ copies/mL/year (Figure 2B), compared to a median SPVL trend of 0.0011 log_10_ copies/mL/year when sampling from years 0 to 100. (95% confidence intervals for the empirical trends are shown overlaid on null distributions created from model years 10–40 in Figure S6B.)

### The affect of sampling biases on empirical SPVL trends

**Figure 3:**
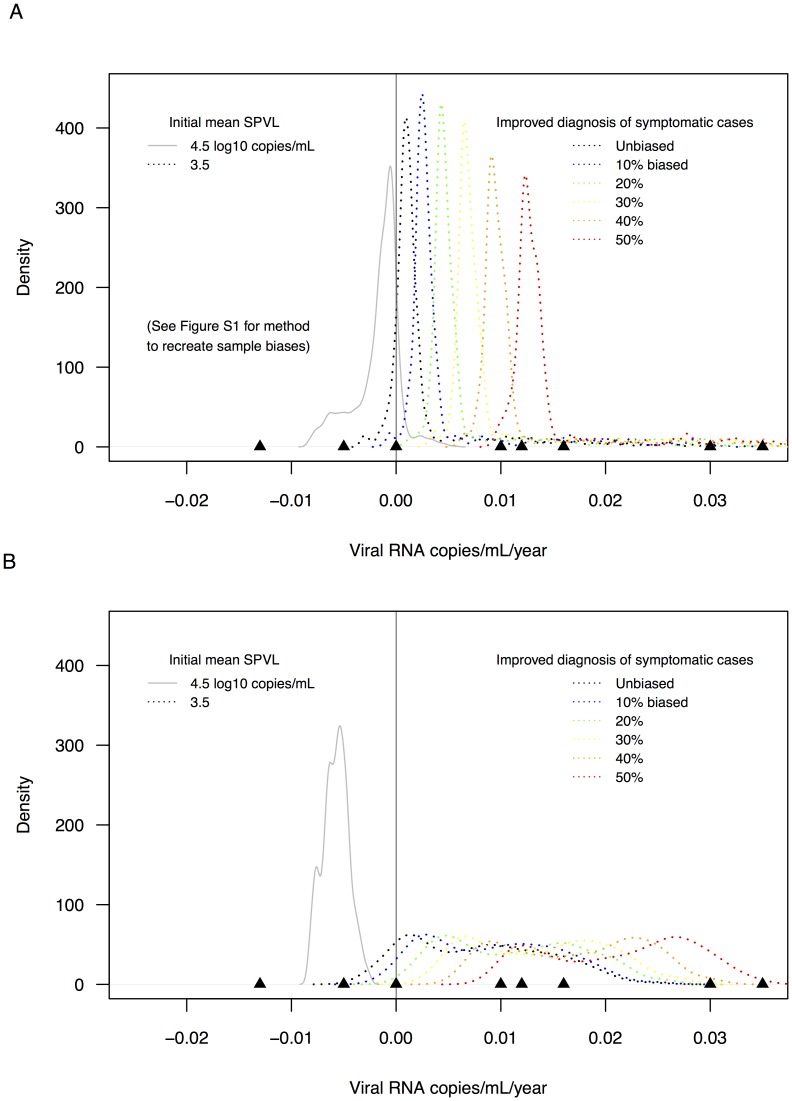
The effect of sampling biases on the estimation of model-based SPVL trends. **A.** Comparison of distribution of 20-year linear SPVL trends estimated from unbiased (black lines, initial mean SPVL = 3.5; grey lines, initial mean SPVL = 4.5) and biased (dotted lines, multiple colors representing multiple sub-sampling levels) data sets. The underlying distributions are produced from years 0 to 100 from simulated epidemics. Removing subsets (%) of all individuals (a schematic representation of the biased sampling process is shown in Figure S1A) results in a distribution of linear trends with a median SPVL trend of greater magnitude than the unbiased trends. **B.** Comparison of distribution of 20-year linear SPVL trends estimated from unbiased and biased data sets, but with the underlying distributions produced from years 10 to 40 from simulated epidemics.

We simulated two types of sampling biases to begin in the latter half of 20-year time periods: 1) earlier ART initiation; and 2) increased rates of diagnosis (*without* associated earlier ART initiation). Hypothetically, these sampling biases may result in linear SPVL trends that are decreased and increased relative to true trends, respectively. Indeed, simulating these biases on model output resulted in incorrect estimates of the true evolutionary trends in SPVL (Figures 3A and 3B). Figure 3A shows incremental positive shifts in SPVL trend distributions that result from increased sampling biases (by increasing the proportion of the HIV-infected population that is be affected by, in this example, increased rates of diagnosis without associated earlier ART). For this specific example (which included only simulated epidemics with initial mean SPVL = 3.5), the medians of biased SPVL trends were significantly different from the median of the true trend distribution (unbiased median = 0.0011; 10% removed median = 0.0028; 50% removed median = 0.0129 log_10_ copies/mL/year). Similar shifted distributions, though in the opposite direction, were found for biases due to earlier ART initiation.

### A more appropriate null distribution to assess sampling biases

Figure 3A shows the effect of sampling biases on null distributions produced from years 0 to 100 of simulated epidemics. As noted above, a more appropriate null distribution may be a time period from simulated epidemics that reflects the expected sampling times of HIV subtype B epidemics; this can be years 10 to 40 of simulated epidemics, reflecting approximately years 1980 to 2010. When this narrowed time period is used to recreate potential sampling biases, the effects on SPVL trend distributions are distinct than those seen when using the full 100 years of simulated epidemics: the median trend values increase significantly with more increased sampling biases (unbiased median = 0.0073; 10% removed median = 0.0094; 50% removed median = 0.0208 log_10_ copies/mL/year), but all distributions overlapped extensively (Figure 3B). In effect, with this narrow null distribution it is more difficult to distinguish between adaptive viral evolution and sampling biases as possible explanations for the empirical SPVL trends.

### SPVL trends at different epidemic stages (biases due to evolutionary context)

**Figure 4:**
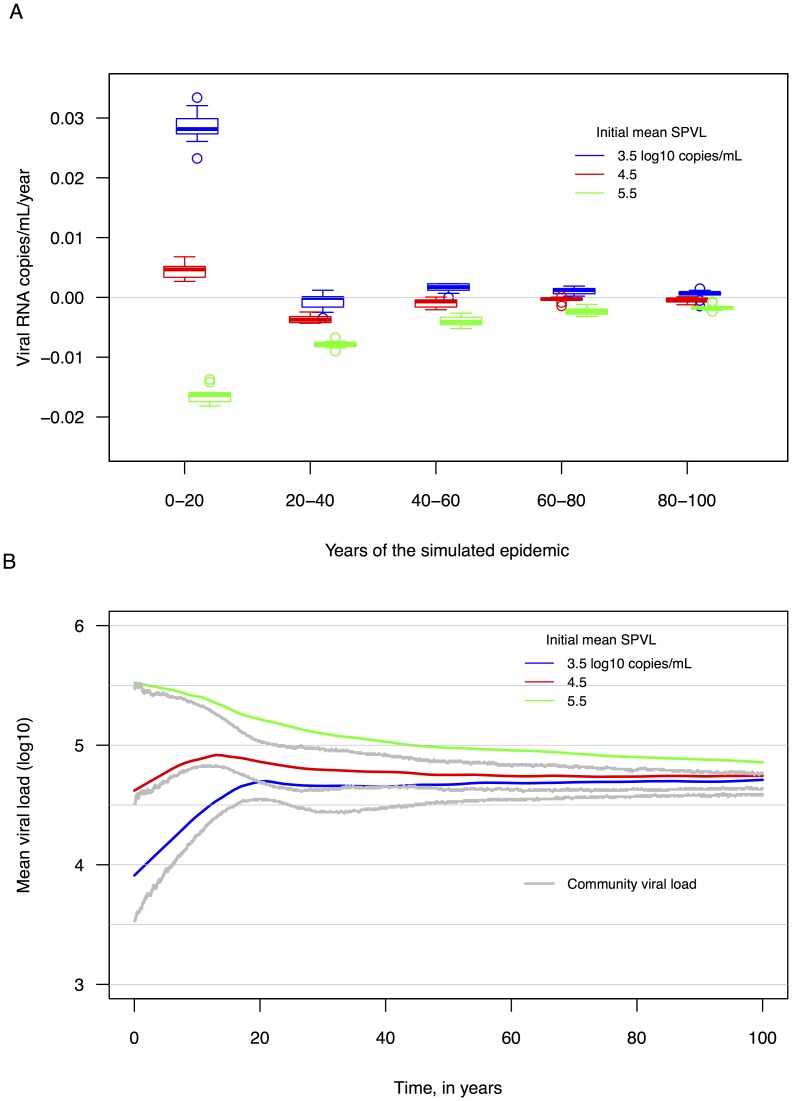
A. Distributions of SPVL trends change as simulated epidemics progress. More extreme SPVL trends occur very early in simulated epidemics (the first 20 years). Boxplots of linear SPVL trends estimated from 100 randomly sampled 20-year time periods; thick line = median; box edges = quartiles; whiskers = minimum and maximum trends. **B.** **Trends in mean community viral load and mean set point viral load are related.** Mean community viral load can evolve over time in the absence of HIV prevention programs. Community viral loads are estimated for each day using viral load measurements from each infected and alive individual, except for those individuals who have been infected less than 45 days (acute infection lasts for 3 months days in these simulations).

An assumption inherent to many studies of HIV virulence is that SPVL trends will be linear. This assumption is likely false, as epidemic growth or decline is affected by the availability of susceptible individuals (among other factors); epidemics may not experience constant linear growth, and thus evolutionary pressures on the virus may shift over the course of an epidemic. Furthermore, processes of HIV evolution, including natural selection and genetic drift, can be affected in complex ways by changes in the viral effective population size (measured in this context most simply as prevalence). We examined the distribution of SPVL slopes in progressing stages of simulated epidemics (Figure 4A). For simulated epidemics with varying initial mean SPVLs (3.5, 4.5, and 5.5 log_10_ copies/mL), the distribution of slopes changed over time, as more extreme slopes (further away from zero) occurred in the first 20 years of epidemics, and all distributions converged to near zero as the epidemic entered years 40 and greater. This suggests that if the empirical SPVL trends are due to adaptive viral evolution, then we can hypothesize that local/regional epidemics (represented by national HIV cohort populations) with increasing SPVL were founded by viruses of low virulence (SPVL∼3.5).

### Sensitivity to parameter values

We tested whether model output was sensitive to the parameters of the viral load functions. We initially focused on: 1) the viral load progression rate in chronic infection; 2) the maximum rate of progression to AIDS; and, 3) the maximum daily rate of transmission in the asymptomatic period. Variation in the rate of viral load increase and in the rate of disease progression had only minor effects on epidemic growth and the evolution of virulence (Figures S7 and S8). Variation in the maximum transmission rate (*B*_max_) had large effects on epidemic growth and the early pattern of evolution toward optimal SPVL (Figure S9). With higher maximum transmission rates, the epidemic size increases more rapidly and virulence (SPVL) increases; these increases in SPVL were transient, however, as the supply of susceptible individuals soon declines and limits further epidemic growth. The evolutionarily optimal SPVL remains equivalent across different values of *B_max_*. This pattern of increasing virulence in the early stage of the epidemic followed by decreasing virulence as susceptible supply declines might be expected [35] and was also observed in the simulations of Shirreff [13].

Model simulations with *B*_max_ following directly from Fraser [12] (*B*_max_ = 0.001044 per day) resulted in epidemic growth that was slow relative to expectations from empirical HIV epidemic data. As *B*_max_ in Fraser [12] and Shirreff [13] was the maximum transmission rate estimated for serodiscordant couples within HIV cohorts [9], and likely an underestimate of the transmission rate in the general population [12], we increased *B*_max_ for our standard runs (*e.g.*, Figures 1 to S7) to *B*_max_ = 0.0025 per day. Increasing *B*_max_ allowed more accurate recreation of HIV epidemic growth rates; this did not affect the optimal HIV SPVL, but variation in *B*_max_ did affect the rate at which SPVL changes in the early epidemic, with higher *B*_max_ associated with early increases to higher SPVL (Figure S9). In addition to the viral load progression rate, the maximum rate of disease progression, and the maximum transmission rate, we examined the effects of variation in other model parameters on SPVL trends. However, the time to peak viremia, the peak viral load in acute infection, the length of acute infection, the viral load at AIDS, the heritability of SPVL at time zero, and the mutational variance of SPVL all had minimal impacts on optimal SPVL.

### Community viral load trends

In our simulated epidemics the mean CVL, measured as the mean or median of log_10_-transformed VL of all infected (and sampled) individuals in a population at a given time, changed over time (Figure 4B). (Mean and median CVL are nearly identical in our simulations.) Community viral load evolved in association with SPVL: CVL trends were influenced by the initial mean SPVL of the founding population, and annual trends in mean/median CVL were qualitatively similar to annual SPVL trends. Mean CVL was consistently lower than mean SPVL due to frailty bias (individuals with low SPVLs were included in CVL estimates more often than individuals with high SPVLs) (Figure 4B). The variance around the mean CVL decreased over the first half of the model runs before stabilizing around 0.3 log_10_ copies/mL. The distribution of all viral loads in our simulations was qualitatively similar to the expected distribution proposed by Miller [22] for a population sample of “detectable viral loads from individuals not on treatment.”

**Figure 5:**
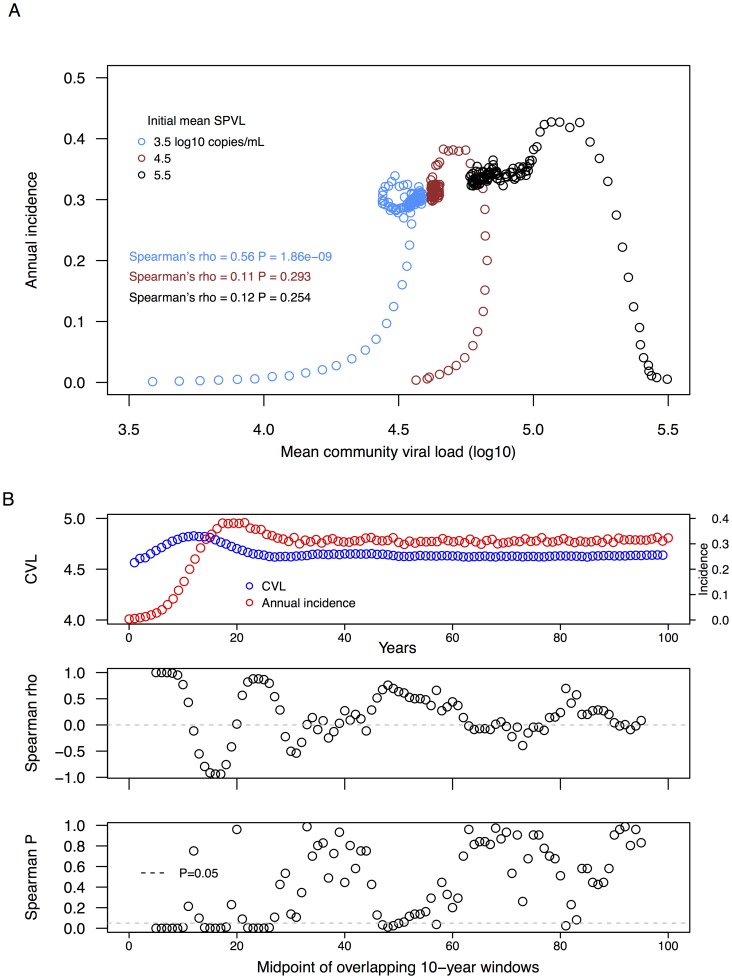
Mean community viral load is not linearly or consistently associated with annual incidence. **A.** Plot of yearly estimates of mean community viral load versus annual incidence, for 100 years of a simulated epidemic. **B.** Distributions of *P*-values for Spearman correlations between mean community viral load and incidence, by year, for 10-year periods from the same 100 year epidemic, from a sliding window of 10 with one year increments. Shown are a plot of CVL and incidence for a 100-year simulated epidemic, Spearman correlation coefficients between CVL and incidence for each overlapping 10-year period, and *P*-value for each Spearman correlation test. Significant associations between CVL and incidence can be positive or negative, depending on epidemic context.

We assessed the hypothesis that CVL is a heuristic measure of a population's overall transmission potential, *i.e.*, that mean CVL is positively associated with incidence [18]–[20]. First, for complete 100-year epidemic simulations, with initial mean SPVL values of 3.5, 4.5 and 5.5 log_10_ copies/mL, we compared yearly values of mean CVL to annual incidence (infected/susceptible) for each year (Figure 5A). Over this extended timescale, mean CVL and annual new infections were significantly correlated only for initial SPVL = 3.5 (Spearman's rho = 0.56, *P*-value = 1.86^−^09; for initial SPVL = 4.5, rho = 0.11, *P*-value = 0.293; for initial SPVL = 5.5, rho = 0.12; *P*-value = 0.254).

Next, we modified this model-based comparison of CVL and incidence in order to make comparisons more equivalent to the 10-year timescales of empirical observations of CVL [18]–[20]. We created a data set containing all 10-year time periods from the 100-year simulated epidemics, each created with a sliding window of length 10 years with increment of one year. For each of these 10-year periods, we estimated Spearman's rho and *P*-value between yearly CVL and new infections (Figure 5B). A minority of 10-year time periods from each epidemic (each initial mean SPVL) contained significant (*P*<0.05) associations between yearly mean CVL and number of infections, and the direction of association (Spearman's rho) alternated between positive and negative over the course of the epidemic (Figure 5B). These simulations were run in the absence of prevention programs such as antiretroviral therapy, yet suggest that mean CVL is not necessarily a strong predictor of HIV transmission potential.

## Discussion

We developed a stochastic agent-based HIV evolutionary and epidemic model that allows for the virulence phenotype, defined by the set point viral load, to change over time. The model is based on viral dynamic functions of transmission, disease progression, and heritability, but retains standard HIV epidemic model output such as prevalence, incidence and the proportion of transmissions occurring during different stages of infection. With this model we addressed questions related to HIV virulence evolution, sampling biases, and epidemic context, and explored how viral dynamic parameters affect SPVL trends and epidemic growth. We also addressed questions about the relationship between community viral load and incidence, in order to assess the use of community viral load as a useful public health metric.

### Set point viral load trends

Our model shows that HIV virulence, using set point viral load as a proxy, can adaptively evolve in a host population. This result is consistent with previous work [12], [13], in both evolutionary processes (adaptation of HIV virulence to optimize transmission potential) and patterns (optimal SPVL ∼4.75 log_10_ copies/mL). It is also consistent with studies showing HIV adaptation to the human population in response to both cellular [36], [37] and humoral [38], [39] responses over the course of the epidemic.

To assess the published empirical trends in SPVL, we created hypothetical null distributions of 20-year linear trends in SPVL and placed the empirical trends within these distributions. Because local epidemics with published SPVL trends may have unique epidemic and evolutionary contexts, particularly the SPVL (virulence) of the founding viral strain, we created null distributions of SPVL trends for simulated epidemics with initial SPVL means of 3.5, 4.5 and 5.5 log_10_ copies/mL. Notably, the simulated and empirical trends are within the same magnitude (between −0.02 to 0.03 log_10_ copies/mL/year), which suggests that adaptive HIV evolution may explain observed trends in HIV virulence.

What explains the variation among empirical trends? The placement of the empirical SPVL trends spans the three null distributions; yet, five (out of eight) lie within the upper distribution (>0.01 log_10_ copies/mL/year) of the simulated trends (Figures 2A). If we narrow our null distribution to years 10 to 40 of simulated epidemics to better reflect the years 1980 to 2010 of European and North American subtype B epidemics, the empirical trends of greater magnitude appear more consistent with model-based trends; these empirical trends are consistent with these epidemics being founded by viral populations of low virulence (virulence less than the optimal level) (Figure 2B). We know from the respective publications that 4 of these 5 empirical trends belong to HIV cohorts with initial mean or median SPVLs less than our model-predicted optimal SPVL (<∼4.75): 3.6 [40], 4.19 [41], 4.3 [42], and 4.4 [43] log_10_ copies/mL (the first sampling period for these studies was most often a 2 to 5-year period starting in 1985; this would correspond to a time approximately between years 15 to 20 in our simulated epidemics). It is essential for future work to compare simulated adaptive evolution of SPVL to real epidemic data, using phylodynamic analysis to reconstruct epidemic histories overlaid with empirical and simulated SPVL trends.

### Recreation of cohort-specific sampling biases

While our model results suggest that adaptive evolution may explain empirical trends, and that epidemic context may explain variation in empirical trends, we also assessed whether trends and variation among trends could be the result of cohort-specific sampling biases. To do so, we recreated two types of simple biases: improvements in diagnosis that result in increased sampling of high virulence individuals; or earlier ART initiations that result in decreased sampling of high virulence individuals. We found that the empirical SPVL trends are consistent with biased trends resulting from improved diagnosis of symptomatic cases. Whether this particular type of sampling bias may explain the empirical trends—as opposed to being explained by adaptive evolution—requires study of specific cohort clinical and community practices. Diagnosis of symptomatic individuals without earlier ART initiation is not consistent with clinical practice; it is unclear what the strength of this bias could be over time in HIV cohorts, and it is likely that both of the potential biases recreated here do exist (to some relative degree) in every HIV cohort.

We can try to infer, using data from the meta-analysis of observed SPVL trends [11] and our model output, which potential type of sampling bias is stronger in the empirical data. The meta-analysis contained 8 studies of SPVL trends, 6 of which were prospective cohorts that estimated virulence trends using only SPVL data from individuals with an estimated date of HIV infection. These *seroconverter cohorts* are less vulnerable to sampling biases, because individuals enter the cohort uninfected. In the meta-analysis, the summary SPVL trend for all eight cohorts (including both seroconverter and seroprevalent cohorts) was 0.013 log_10_ copies/mL/year, 38% lower than the summary trend of 0.018 seen for the six seroconverter cohorts. A discrepancy between trend estimates in this direction (decreasing SPVL trend) is consistent with a sampling bias caused by earlier ART initiation in the seroprevalent cohorts. (*i.e.* the lower SPVL trends in the seroprevalent cohorts could be explained by a sampling bias caused by earlier ART initiation in those populations). This hypothesis is consistent with decreasing clinical thresholds for ART initiation (http://aidsinfo.nih.gov/guidelines/archive/adult-and-adolescent-guidelines).

### Community viral load trends

The use of community viral load as a population-level metric of HIV transmission potential has been proposed [18]–[21]. To date few studies, empirical or modeling, have assessed this proposition thoroughly [22]; for CVL to be a useful tool for public health inferences, it must accurately and precisely reflect HIV epidemiological parameters of interest. There are similarities between the estimation of SPVL and CVL trends—we hypothesize that understanding the underlying causes or sampling biases related to SPVL trends can inform our understanding of CVL trends. With our model, we attempted to evaluate the possible affects of HIV epidemic and evolutionary context on trends in mean CVL. Our findings suggest the relationship between CVL and incidence is not straightforward, yet is strongly modulated by epidemic context, including: 1) the initial mean SPVL of an epidemic; and 2) the epidemic stage in which the CVL and incidence relationship is evaluated. As shown in Figure 5B, significant associations between CVL and incidence can be identified in simulated epidemics, but there are both positive and negative associations. In this scenario CVL is not a robust population-level metric of HIV transmission potential. However, our estimate of CVL does not include individuals on ART with depressed viral loads and thus does not perfectly coincide with real world applications. Nevertheless, our model shows that attempts to infer transmission potential from CVL can give highly misleading results, as CVL is influenced by both historical and evolutionary factors.

### Caveats and future additions

The global HIV pandemic is one of multiple separate epidemics that can be stratified by viral genotype (subtypes or circulating recombinant forms) [44], [45], and by human population (*e.g.*, transmission risk group, geography) [46], [47]. Our model is not designed to recapitulate the entire global pandemic, but rather local epidemics containing a single subtype. The disease progression function of our model is based on subtype B data; it is possible that the relationship between SPVL and disease progression is different across subtypes [48]–[52], but the disease progression function likely holds for different risk groups within local subtype B epidemics (*e.g.*, heterosexual sex, men who have sex with men, or injecting drug use within subtype B epidemics). The transmission function of our model is based on serodiscordant heterosexual couples with counseling [9]; as discussed above, using *B*_max_ from Fraser [12] that saturates at viral loads results in slower epidemic growth rates. Elevating this rate to perhaps better reflect transmission rates in the general population results in more realistic growth rates but a similar level of optimal virulence. Additionally, SPVL is known to vary among hosts due to host genetics (HLA type) [53]; our model does not distinguish among individuals in their susceptibility to infection or host effect on SPVL (which can influence transmission and disease progression). Nor does our model allow for variation within individuals in viral reproductive rate; *i.e.*, in our model all viral lineages within a single person are assumed to contain the same viral genetic factors for viral reproduction and SPVL/virulence. When this is not the case, and virions within a host are allowed to vary in reproductive rate, it is theoretically possible that different evolutionary trends in relation to optimal SPVL may be seen [54].

Our model includes simple demographic and sexual mixing terms, which we believe are sufficient to address the issues explored in this paper. A notable result from our model epidemics was the relatively low frequency of transmissions from “early HIV infection” relative to other published estimates [55]. For our standard epidemic runs, ∼10% to 25% of infections took place while the source partner was in “early HIV infection” (with “early HIV infection defined as either the duration of acute infection or the first year of infection, respectively). Further work will clarify whether our low estimates suggest that behavioral or network parameters (rather than strictly viral dynamics, as included in our model) are the likely source of the high contribution of early HIV infection to onward transmission that is reported elsewhere. An additional possible cause of the high reported frequencies of transmission in early HIV infection are viral genetic factors associated with increased transmissibility in early infection [56]–[59]; our model does not provide viruses with stage-specific transmission probabilities.

We plan to extend our model and analysis by comparing virulence trends among populations with more complex sexual mixing patterns, and among populations with varying sample fractions at different stages of an ART treatment cascade. We hope this epidemic modeling approach based on viral dynamics will be a useful tool in the prediction or evaluation of potential outcomes of prevention programs.

## Supporting Information

Figure S1 **Recreation of simple SPVL sampling biases.** The schematic illustrates two types of potential sampling biases, each reconstructed within a 20-year period (randomly-selected from a larger 100-year epidemic). Each 20-year period is divided into two 10-year periods, with the second 10-year period sub-sampled to recreate a sampling bias that may occur as an HIV epidemic progresses within a clinic, cohort, region or country. The blue region illustrates a sampling bias caused by improved diagnosis of symptomatic cases in the 2^nd^ 10-year period (symptomatic cases are associated with higher viral loads; SPVLs>5.0 log_10_ copies/mL). In this case, one will not sample a greater portion of individuals with low set point viral loads as the epidemic progresses, which we simulate by removing a portion of the individuals with SPVL<5.0 log_10_ copies/mL in the 2^nd^ 10-year period (light blue circles). This would lead to an higher estimated rate of SPVL change over time. The green region illustrates a sampling bias caused by increased rates of earlier ART initiation to symptomatic individuals in the 2^nd^ 10-year period. In this case there are fewer people with higher SPVLs to sample, which we simulate by removing a portion of the individuals with SPVL>5.0 log_10_ copies/mL in the 2^nd^ 10-year period (light green circles). This would lead to an lower estimated rate of SPVL change over time.
(TIF)

Figure S2 **Kaplan-Meier survival curves for quartiles of SPVL.**
(TIF)

Figure S3 **Heritability of SPVL between source and recipient transmission pairs.** Linear regression coefficients of donor and recipient SPVLs estimated for each year of a 100-year simulated epidemic (initial mean SPVL = 4.5 and initial user-defined heritability parameter *h* ^2^ = 0.5 (Equation 6)). Estimates of SPVL heritability decrease over the course of the epidemic, as expected with decreasing variance in SPVL. Inset plot is recipient SPVL by source SPVL at year 40 of a simulated epidemic.
(TIF)

Figure S4 **Example of set point viral load change over time.** Initial (founder) set point viral load is 4.5 log_10_ copies/mL. Smoothed lined represents mean SPVL over time, using a locally weighted polynomial regression curve (Lowess fit = 0.1).
(TIF)

Figure S5 **Frequency of transmission by stage of infection, by epidemic stage.** Frequency of transmissions that occur when the transmitter is in early HIV infection, with early infection defined as either acute infection (3 months in our standard model runs) or the first year after infection.
(TIF)

Figure S6 **Confidence intervals of the empirical SPVL trends.** **A.** Confidence intervals are placed on top of model-produced null distributions produced using years 0 to 100 of the simulated epidemics. Shown are null distributions from multiple runs with initial population mean SPVLs of 3.5, 4.5 and 5.5 log_10_ copies/mL (see Figure 2A). **B.** Confidence intervals are placed on top of model-produced null distributions produced using years 10 to 40 of the simulated epidemics. Shown are null distributions from multiple runs with initial population mean SPVLs of 3.5, 4.5 and 5.5 log_10_ copies/mL (see Figure 2B).
(TIF)

Figure S7 **Viral load increase rate and epidemic growth and set point change.** **A.** Infected individuals and **B.** Mean set point viral load over time. Variation in the annual rate of viral load increase (natural log) show only minor effects on epidemic growth or evolutionarily optimal set point viral load.
(TIF)

Figure S8 **Increased disease progression rates result in increased epidemic growth and decreased optimal set point viral load.** **A.** Infected individuals and **B.** Mean set point viral load over time. Variation in the maximum rate of disease progression (*D*_max_ from Equation 2) from initial infection to AIDS significantly affects epidemic growth and evolutionarily optimal set point viral load.
(TIF)

Figure S9 **Increased viral transmission rates result in increased epidemic growth but similar set point dynamics.** **A.** Infected individuals and **B.** Mean set point viral load over time. Variation in the annual rate of transmission (*B*_max_ in Equation 3) significantly affects epidemic growth (due to changes in the number of susceptible individuals), but does not affect the evolutionarily optimal set point viral load (although it changes the shape of SPVL change).
(TIF)

Text S1 **Additional detailed description of the model.**
(DOCX)
