# Supplementary figures and images for: An HIV Epidemic Model Based on Viral Load Dynamics: Value in Assessing Empirical Trends in HIV Virulence and Community Viral Load
Source: PLoS Comput Biol. 2014 Jun 19;10(6):e1003673. doi: 10.1371/journal.pcbi.1003673 (PMC4063664; doi:10.1371/journal.pcbi.1003673)

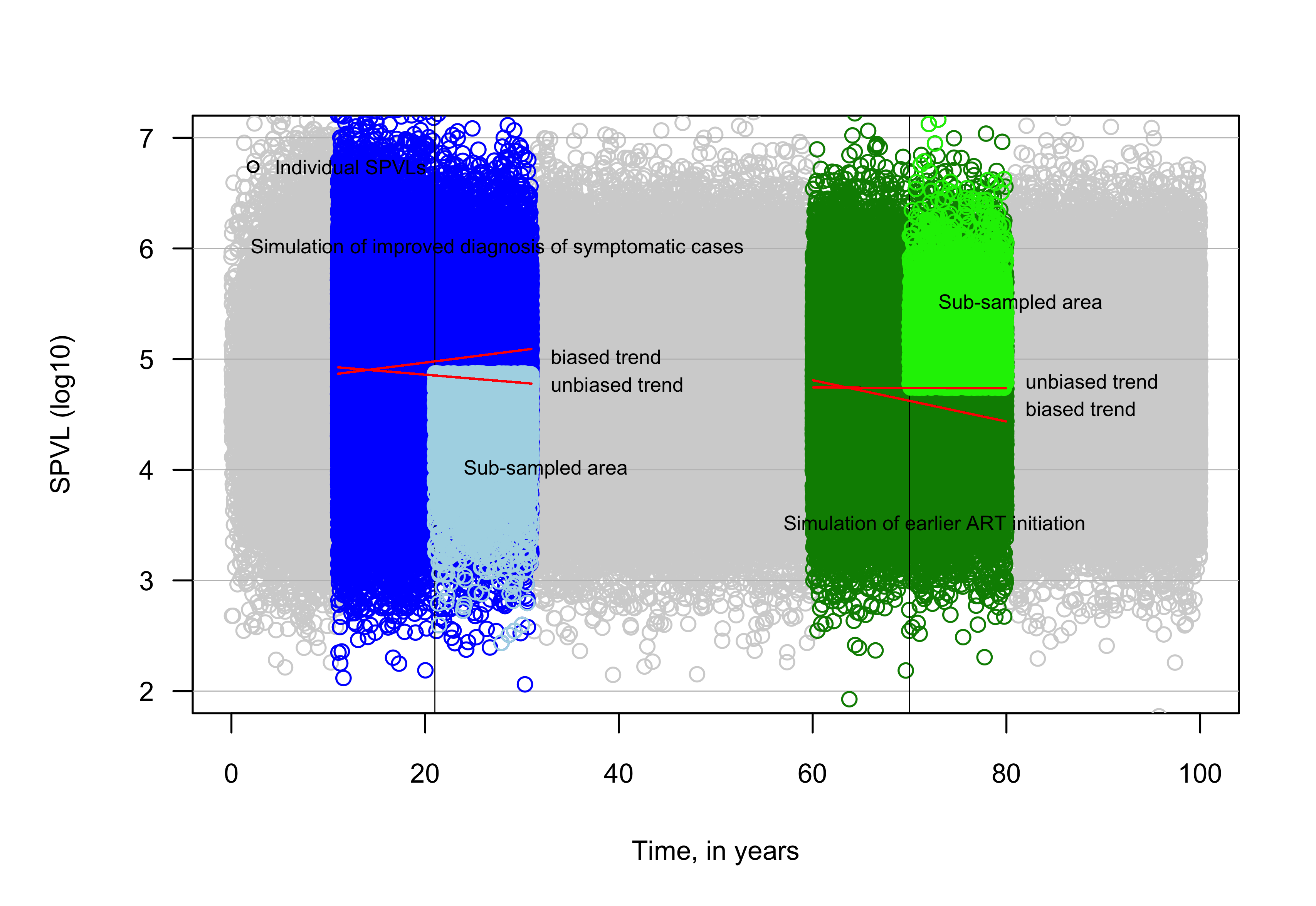

Supplement: Figure S1 — Recreation of simple SPVL sampling biases. The schematic illustrates two types of potential sampling biases, each reconstructed within a 20-year period (randomly-selected from a larger 100-year epidemic). Each 20-year period is divided into two 10-year periods, with the second 10-year period sub-sampled to recreate a sampling bias that may occur as an HIV epidemic progresses within a clinic, cohort, region or country. The blue region illustrates a sampling bias caused by improved diagnosis of symptomatic cases in the 2nd 10-year period (symptomatic cases are associated with higher viral loads; SPVLs>5.0 log10 copies/mL). In this case, one will not sample a greater portion of individuals with low set point viral loads as the epidemic progresses, which we simulate by removing a portion of the individuals with SPVL<5.0 log10 copies/mL in the 2nd 10-year period (light blue circles). This would lead to an higher estimated rate of SPVL change over time. The green region illustrates a sampling bias caused by increased rates of earlier ART initiation to symptomatic individuals in the 2nd 10-year period. In this case there are fewer people with higher SPVLs to sample, which we simulate by removing a portion of the individuals with SPVL>5.0 log10 copies/mL in the 2nd 10-year period (light green circles). This would lead to an lower estimated rate of SPVL change over time. (TIF) [file pcbi.1003673.s001.tif]

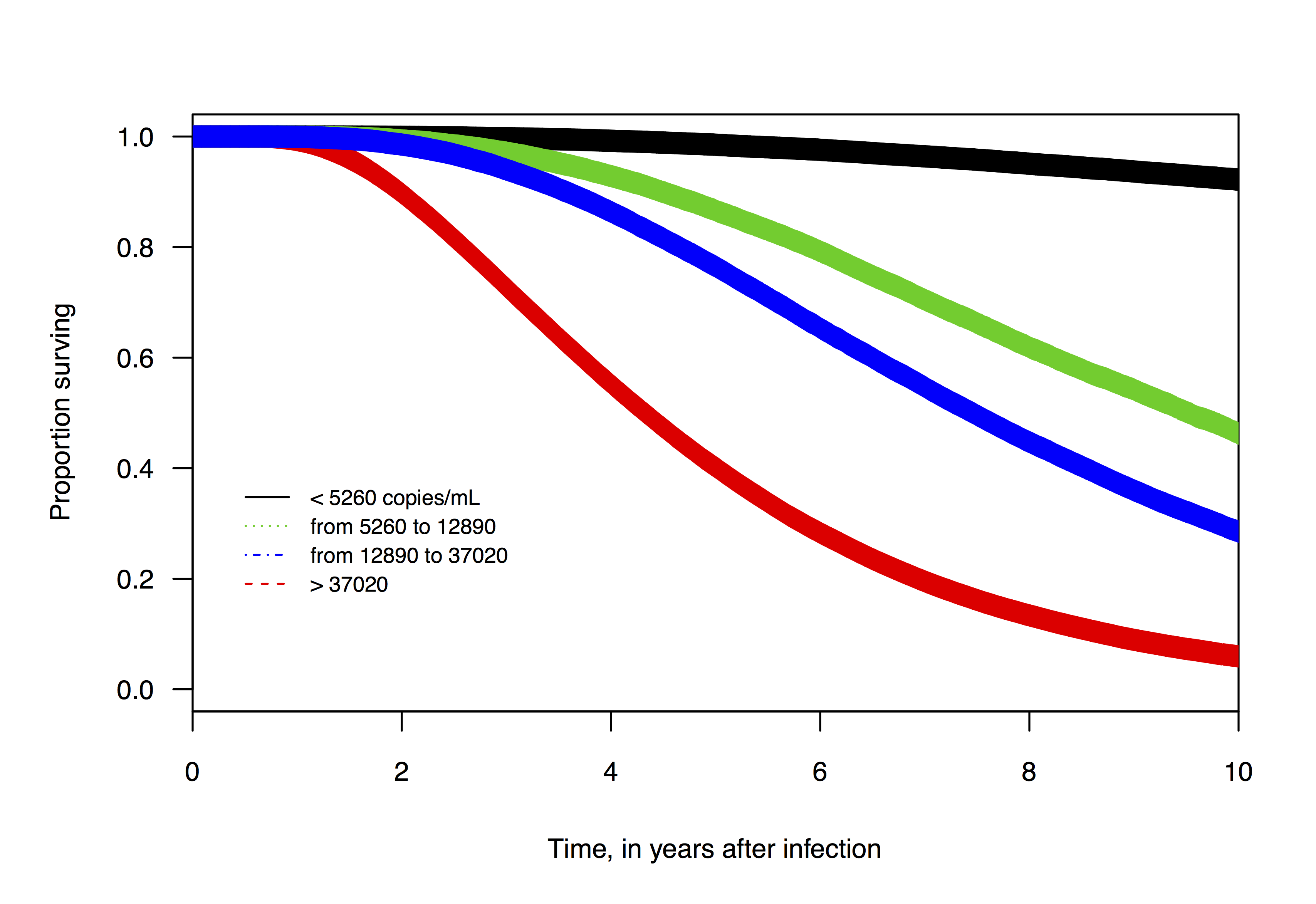

Supplement: Figure S2 — Kaplan-Meier survival curves for quartiles of SPVL. (TIF) [file pcbi.1003673.s002.tif]

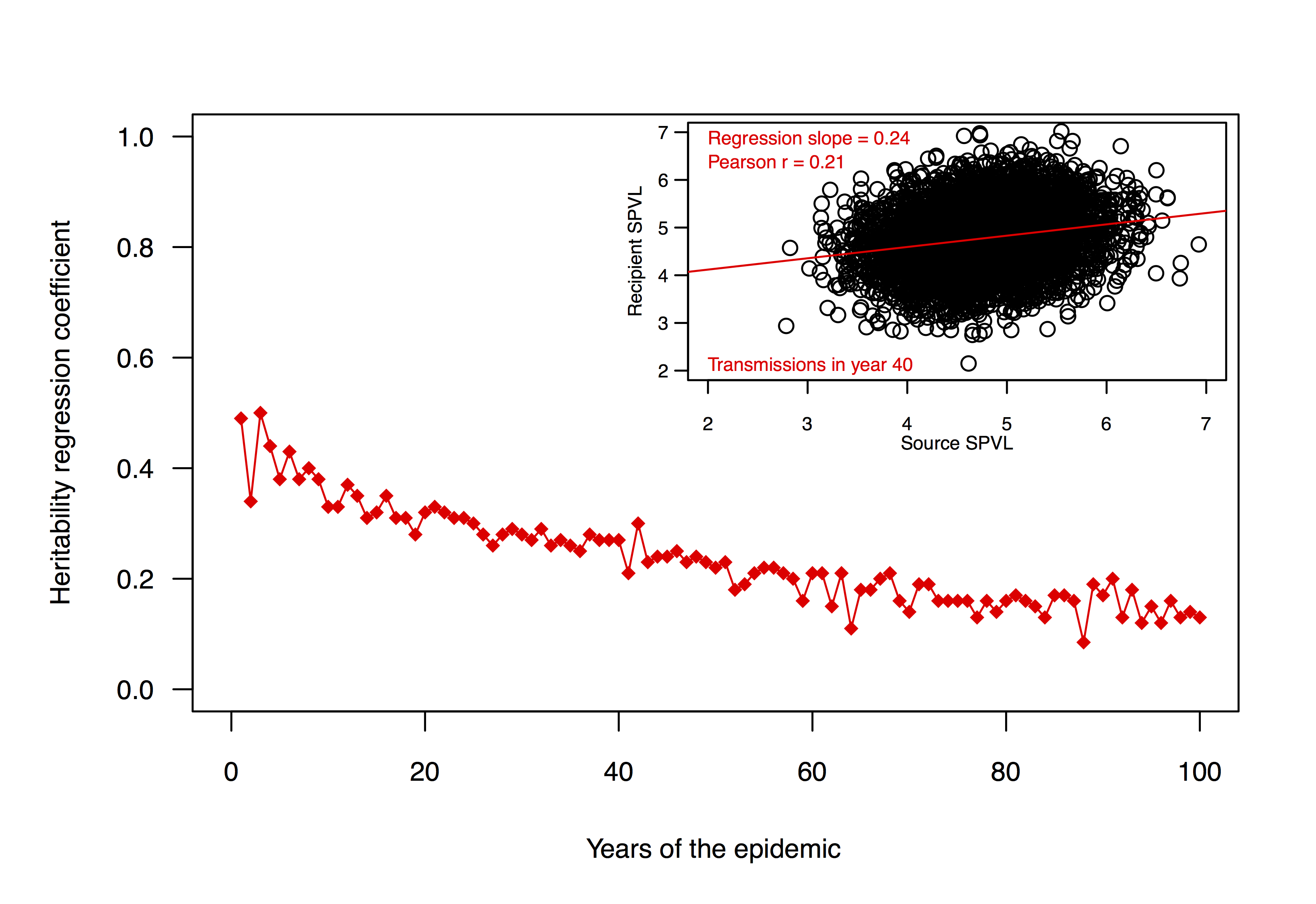

Supplement: Figure S3 — Heritability of SPVL between source and recipient transmission pairs. Linear regression coefficients of donor and recipient SPVLs estimated for each year of a 100-year simulated epidemic (initial mean SPVL = 4.5 and initial user-defined heritability parameter h 2 = 0.5 (Equation 6)). Estimates of SPVL heritability decrease over the course of the epidemic, as expected with decreasing variance in SPVL. Inset plot is recipient SPVL by source SPVL at year 40 of a simulated epidemic. (TIF) [file pcbi.1003673.s003.tif]

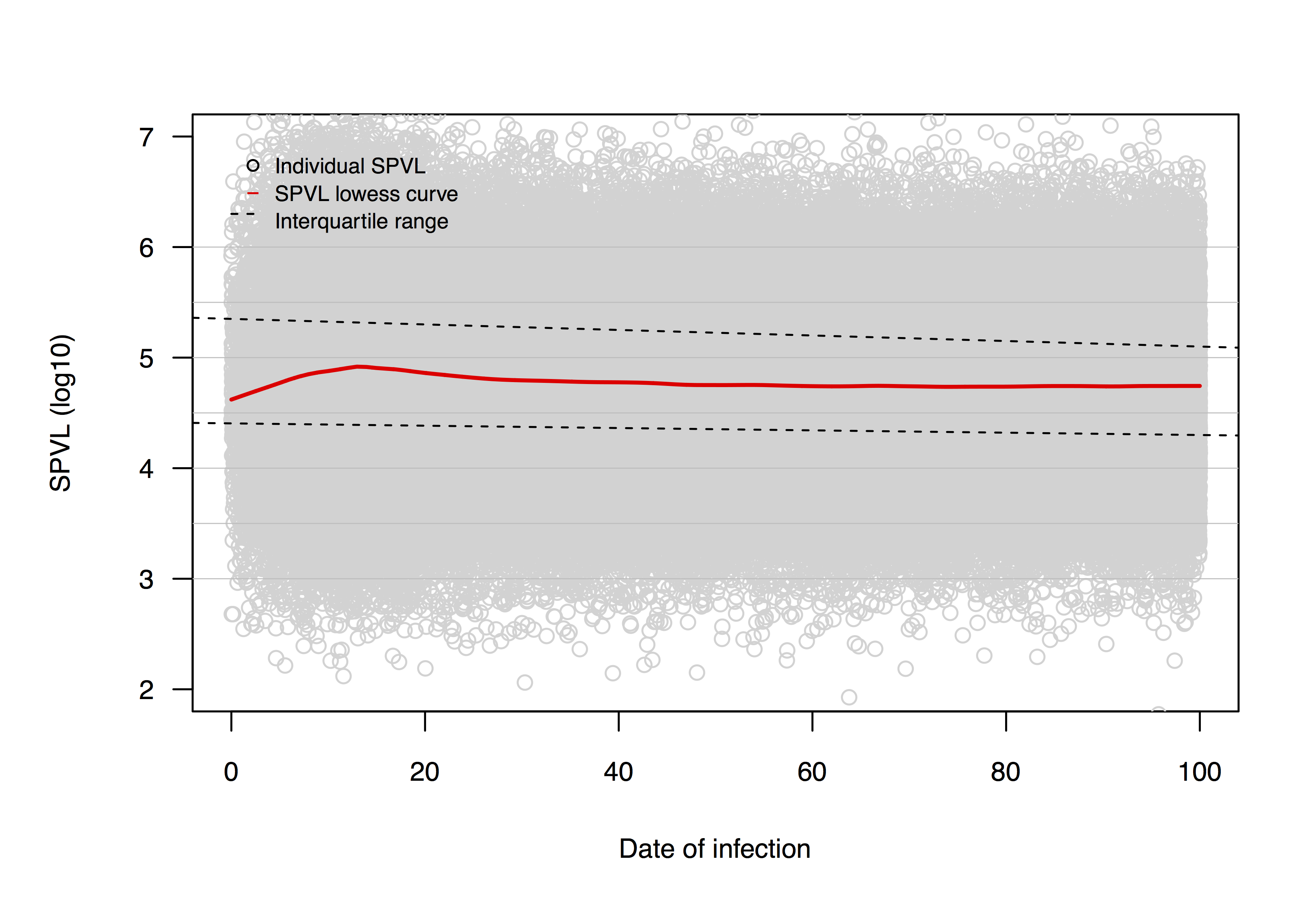

Supplement: Figure S4 — Example of set point viral load change over time. Initial (founder) set point viral load is 4.5 log10 copies/mL. Smoothed lined represents mean SPVL over time, using a locally weighted polynomial regression curve (Lowess fit = 0.1). (TIF) [file pcbi.1003673.s004.tif]

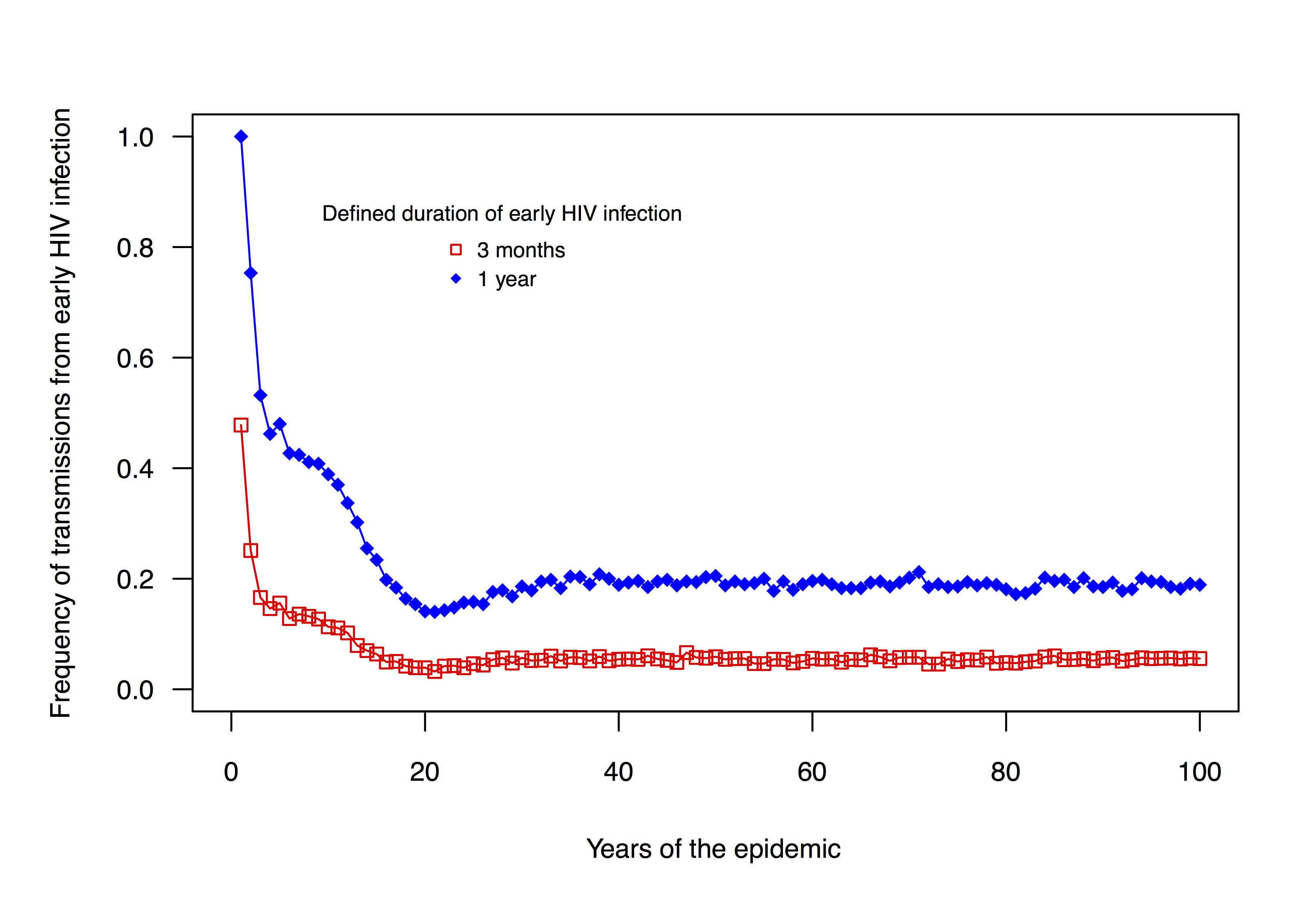

Supplement: Figure S5 — Frequency of transmission by stage of infection, by epidemic stage. Frequency of transmissions that occur when the transmitter is in early HIV infection, with early infection defined as either acute infection (3 months in our standard model runs) or the first year after infection. (TIF) [file pcbi.1003673.s005.tif]

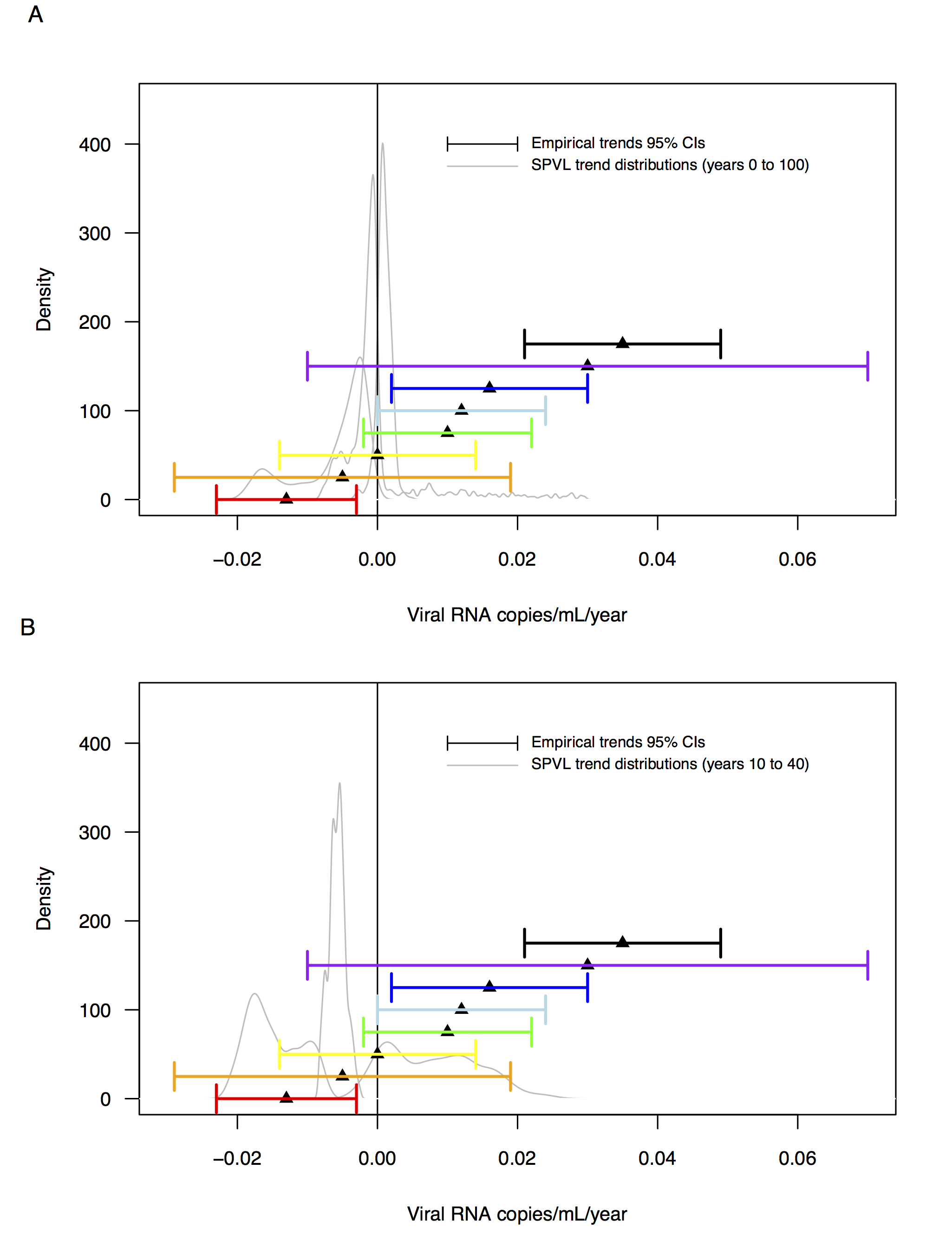

Supplement: Figure S6 — Confidence intervals of the empirical SPVL trends. A. Confidence intervals are placed on top of model-produced null distributions produced using years 0 to 100 of the simulated epidemics. Shown are null distributions from multiple runs with initial population mean SPVLs of 3.5, 4.5 and 5.5 log10 copies/mL (see Figure 2A). B. Confidence intervals are placed on top of model-produced null distributions produced using years 10 to 40 of the simulated epidemics. Shown are null distributions from multiple runs with initial population mean SPVLs of 3.5, 4.5 and 5.5 log10 copies/mL (see Figure 2B). (TIF) [file pcbi.1003673.s006.tif]

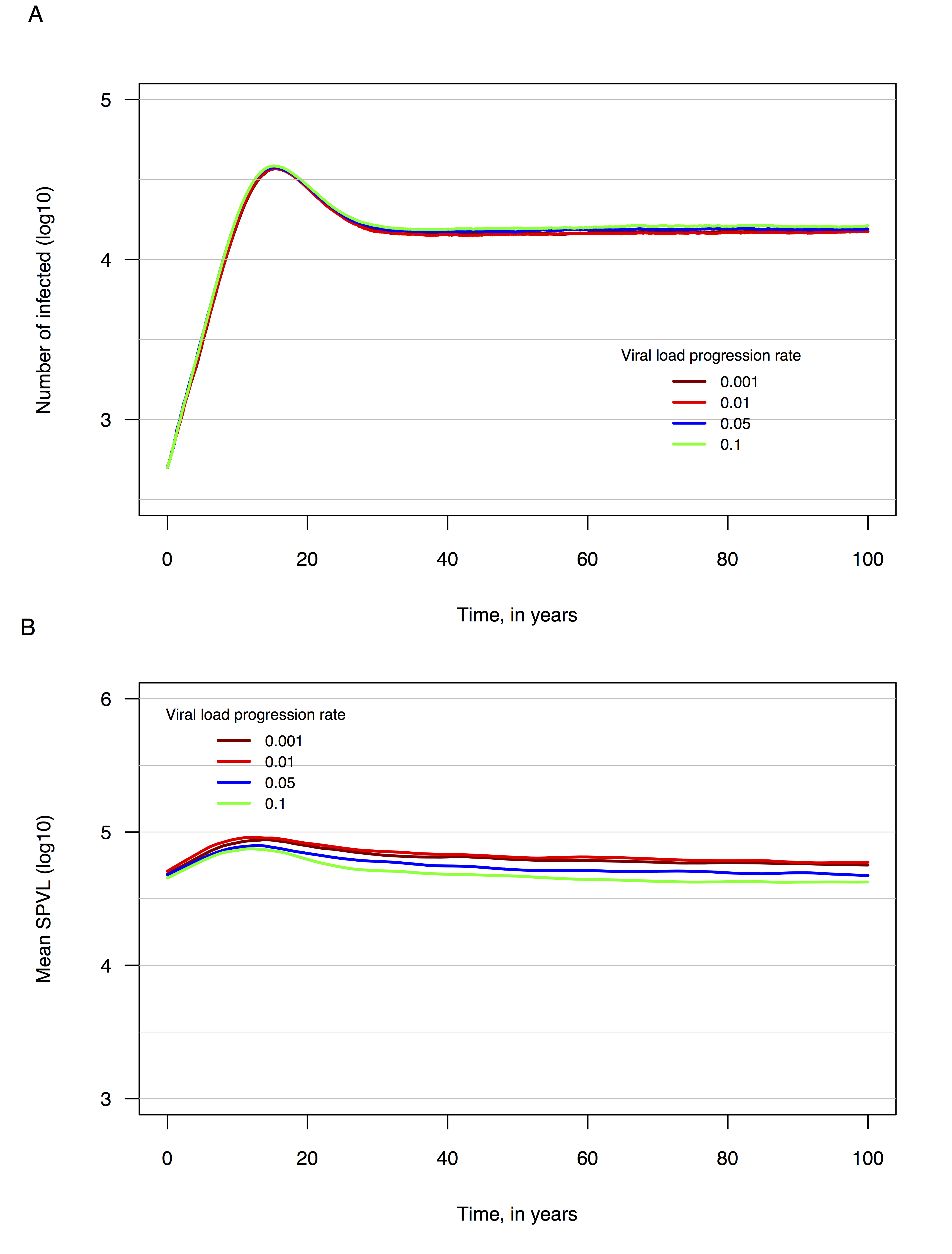

Supplement: Figure S7 — Viral load increase rate and epidemic growth and set point change. A. Infected individuals and B. Mean set point viral load over time. Variation in the annual rate of viral load increase (natural log) show only minor effects on epidemic growth or evolutionarily optimal set point viral load. (TIF) [file pcbi.1003673.s007.tif]

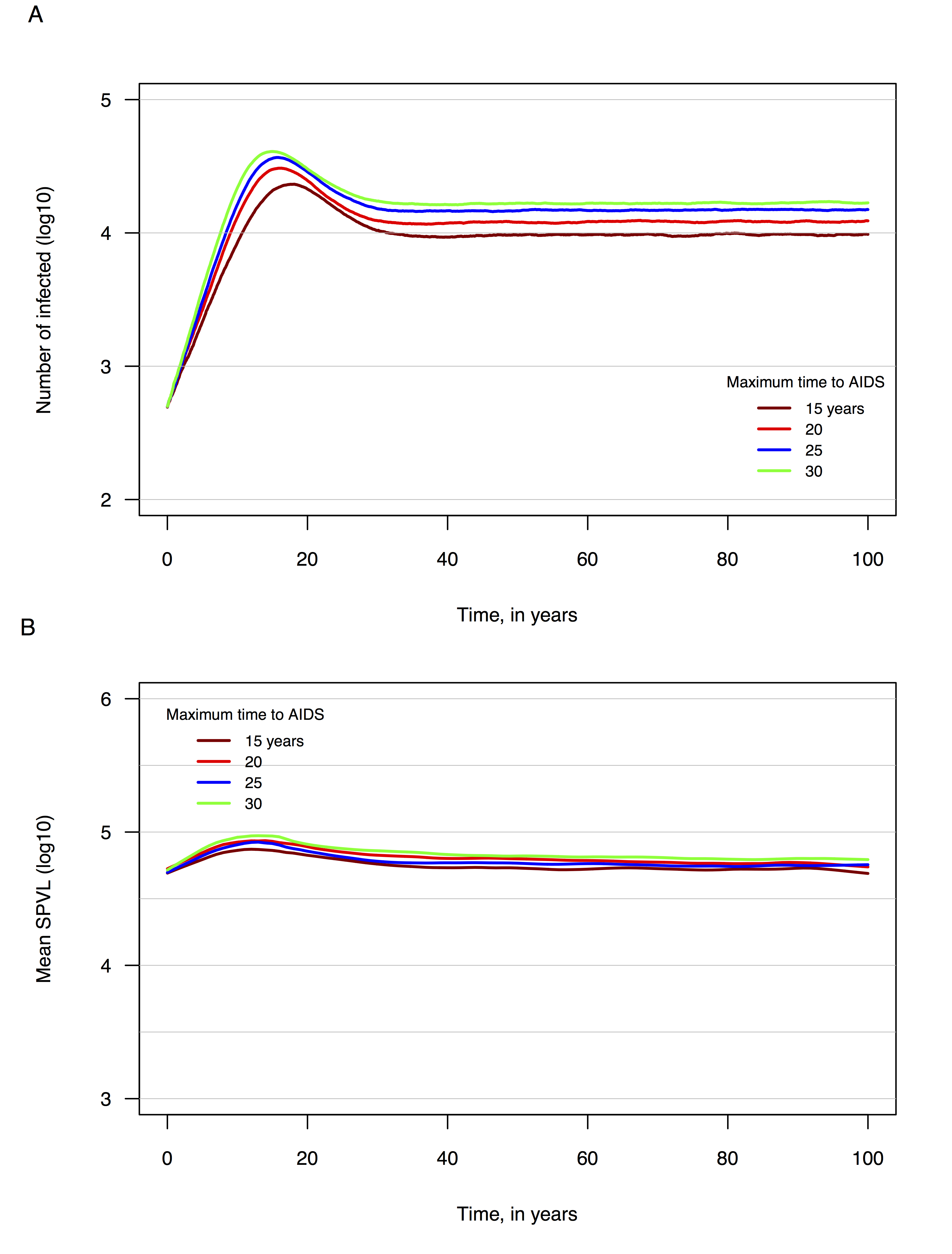

Supplement: Figure S8 — Increased disease progression rates result in increased epidemic growth and decreased optimal set point viral load. A. Infected individuals and B. Mean set point viral load over time. Variation in the maximum rate of disease progression (D max from Equation 2) from initial infection to AIDS significantly affects epidemic growth and evolutionarily optimal set point viral load. (TIF) [file pcbi.1003673.s008.tif]

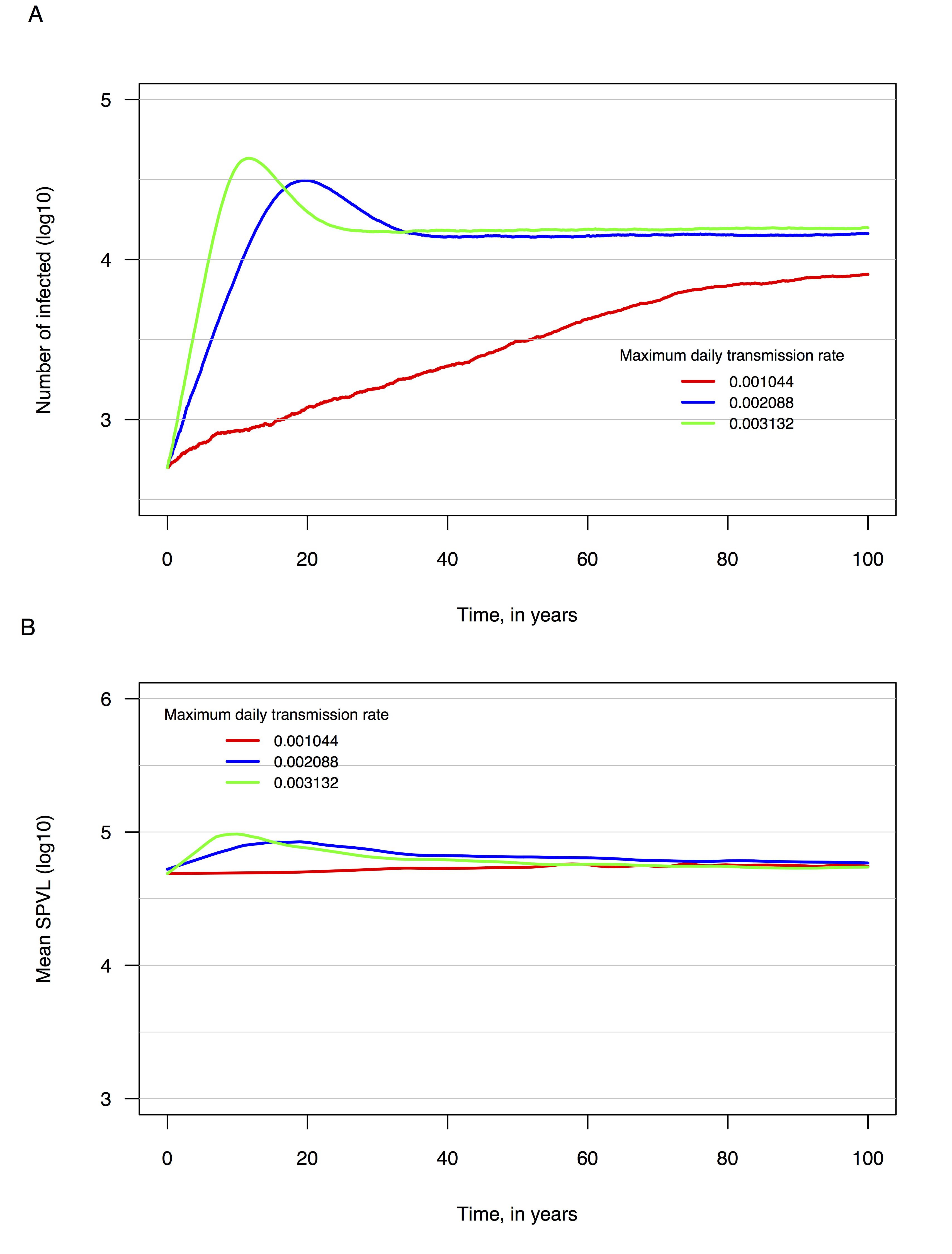

Supplement: Figure S9 — Increased viral transmission rates result in increased epidemic growth but similar set point dynamics. A. Infected individuals and B. Mean set point viral load over time. Variation in the annual rate of transmission (B max in Equation 3) significantly affects epidemic growth (due to changes in the number of susceptible individuals), but does not affect the evolutionarily optimal set point viral load (although it changes the shape of SPVL change). (TIF) [file pcbi.1003673.s009.tif]
