# Supplementary material for: An HIV Epidemic Model Based on Viral Load Dynamics: Value in Assessing Empirical Trends in HIV Virulence and Community Viral Load
Source: PLoS Comput Biol. 2014 Jun 19;10(6):e1003673. doi: 10.1371/journal.pcbi.1003673 (PMC4063664; doi:10.1371/journal.pcbi.1003673)
Supplement: Text S1 — Additional detailed description of the model. (DOCX) [file pcbi.1003673.s010.docx]

**Supporting Text S1. Additional detailed description of the model.**

*Within-host component (viral load dynamics)*

For each individual, upon infection with an initial viral inoculum of size *V*_0_, viral load increases exponentially at rate *r* until a user-defined peak viremia, *V*_peak_, at a user-defined time *t*_peak_. Viral load then declines exponentially at an individual-specific decay rate, *d*_acute_, until it reaches SPVL, *V*_sp_, at time *t*_sp_. We define *t*_sp_, the time when *V*_sp_ is established, in the number of days after infection; this is a user-defined length from initial infection through peak viremia until set point. After reaching set point (*t*_sp_), the viral load increases as (Equation 1):

*V*(*t*) = V_sp_e*^s^*^(^*^t-tsp^*^)^ (1)

where *s* is the user-defined parameter of annual viral load increase rate, and *t* is the time that has elapsed since the patient was first infected. For all HIV-infected individuals in the model, we assume a log linear change in viral load after set point has been established until the onset of AIDS. Functionally this can be set to zero change, equivalent to stable viral load in the asymptomatic stage. Defining a linear rate of viral load progression in asymptomatic infection is not straightforward: different rates have been reported, from stable to a 0.06 or 0.10 log_10_ annual increase [[1-4](#_ENREF_1)]. Viral load upon onset of AIDS is defined as the same for all individuals, and is independent of SPVL. For the current analysis we have defined viral load during the AIDS stage to be half of *t*_peak_, peak viral load in acute infection. To reconcile population variation in *V*_sp_ with primary infection dynamics, we assume that *V*_0_, *r*, *t*_peak_, *t*_sp_ and *s* are the same in all individuals, but that *d*_acute_ = *ln*(*V*_peak_/*V*_sp_)/(*t*_sp_ - *t*_peak_) varies.

*Within-host component (set point viral load and progression to AIDS-related death)*

The probability of HIV-associated mortality is given by an algorithm [[5](#_ENREF_5),[6](#_ENREF_6)] (Equation 2) in which the time to AIDS for each patient is drawn from a gamma distribution with shape parameter *ρ* and a mean time delay given by:

$t=D_{\max}\frac{D_{50}^{h}}{D_{50}^{h}+V_{sp}^{h}}$ (2)

where *D*_max_ is the maximum time to AIDS, *D*_50_ is the SPVL at which the time to AIDS is half its maximal value, and *h* is a Hill Coefficient that determines how quickly τ increases with *V*_setpoint_ when *V*_setpoint_ is close to *D*_50_. To facilitate comparisons with previous work, we follow Fraser and Shirreff [[5](#_ENREF_5),[6](#_ENREF_6)] for *D*_max_, *D*_50_, and *h*, and the duration of the AIDS stage (*t*_sp_, the time of acute infection stage) (Table 1).

*Across-host component (viral load and transmission)*

We assume that transmission rates follow available data from serodiscordant heterosexual partners; the probability of a HIV-infected person will transmit to a HIV-negative person is determined by an increasing Hill function (Equation 3) that follows Fraser [[5](#_ENREF_5)]:

 (3)

where *B*_max_ is the maximum probability of transmission per year, *V* is the donor’s viral load at the time of sexual contact, *a* is a Hill Coefficient that influences the steepness of the response curve, and *K* is the viral load at which the probability of transmission to a susceptible person is *B*_max_/2.

*Across-host component (heritability of set point viral load)*

The SPVL, *V*_sp_, is determined by both viral (the viral genotype, *VirCont*) and environmental (a combination of undefined host and environmental factors, *EnvCont*) factors. Mathematically, we assume, for donor (*i*) and recipient (*j*) (Equations 4 - 6):

 (4)

 (5)

 (6)

where *h* = the square root of *h*^2^, the user-defined heritability, and the *environmental contribution* = the square root of (1-*h*^2^). *VirCont* is different for each individual; HIV-infected individuals inherit the value of *VirCont* from the individual who infected them, with random variance introduced within a separate mutational variance parameter that affects the transmitted SPVL genotype in an increasing function over the course of infection.

*Sexual mixing network*

For each individual in a simulation, the model maintains a list of sexual partnerships and viral transmission pairs. Individuals were not marked by sex, and risks of infection were bidirectional, meaning that both partners have independent probabilities of infecting the other partner that are dependent on individual viral loads. As such, this model may be more appropriate for men-who-have-sex-with-men (MSM) networks; however, since we are focusing on broad trends, qualitative results from this model should also apply to networks of primarily heterosexual transmissions. For our primary analysis we focused on a simple random mixing model with no concurrency and short partnership durations; additional variations of mixing networks can be created by modifying the length of sexual partnerships and the maximum number of concurrent partners.

For any one simulation, prior to the simulation of viral transmission, an initial set of contacts is formed by randomly choosing pairs from the population. The probability of each person entering into this link is set to *e*^-Li^, where *L*_i_ is the number contacts that person *i* has. If this probability was not met (or if *L*_i_ > *MaxLinks*), another partner is selected at random from the population. This process is repeated until the total number of links equals *N***M*/2, where *N* is the total number of sexual active individuals, and *M* is the mean degree. The probability of a connection between individuals *i* and *j* dissolving is set to 2/(*Duration*[*i*] * *Duration*[*j*]), where *Duration*[*x*] is the expected time that person *x* stays in a relationship. After removing links from all newly dissolved partnerships, *N***M*/2 - *L*/2 links are added to the system, where *N* is the number of individuals after accounting for births and deaths that occurred that day and *L* is the number of links in the system after the dissolution step. If this quantity is negative, no links are added.

1. Bonhoeffer S, Funk GA, Gunthard HF, Fischer M, Muller V (2003) Glancing behind virus load variation in HIV-1 infection. Trends Microbiol 11: 499-504.

2. Shankarappa R, Margolick JB, Gange SJ, Rodrigo AG, Upchurch D, et al. (1999) Consistent viral evolutionary changes associated with the progression of human immunodeficiency virus type 1 infection. J Virol 73: 10489-10502.

3. OBrien TR, Rosenberg PS, Yellin F, Goedert JJ (1998) Longitudinal HIV-1 RNA levels in a cohort of homosexual men. J Acquir Immune Defic Syndr Hum Retrovirol 18: 155-161.

4. Geskus RB, Prins M, Hubert JB, Miedema F, Berkhout B, et al. (2007) The HIV RNA setpoint theory revisited. Retrovirology 4: 65.

5. Fraser C, Hollingsworth TD, Chapman R, de Wolf F, Hanage WP (2007) Variation in HIV-1 set-point viral load: epidemiological analysis and an evolutionary hypothesis. Proc Natl Acad Sci U S A 104: 17441-17446.

6. Shirreff G, Pellis L, Laeyendecker O, Fraser C (2011) Transmission selects for HIV-1 strains of intermediate virulence: a modelling approach. PLoS Comput Biol 7: e1002185.
